# Supplementary material for: Upper Normal Serum Creatinine Concentrations as a Predictor for Chronic Kidney Disease: Analysis of 14 Years’ Korean Genome and Epidemiology Study (KoGES)
Source: J Clin Med. 2018 Nov 21;7(11):463. doi: 10.3390/jcm7110463 (PMC6262490; doi:10.3390/jcm7110463)
Supplement: Supplementary file 1 [file jcm-07-00463-s001.pdf]

SUPPLEMENTAL MATERIAL

Supplemental Table 1. Factors associated with the decline rate of eGFR [a,b]

	Female		Male	
	**ß (95% CI)**	***P***	**ß (95% CI)**	***P***
Age (per 1 year)	0.07 (0.06-0.08)	<0.001	0.05 (0.04-0.06)	<0.001
Muscle mass (per 1kg)	0.00 (-0.03-0.03)	0.98	-0.03 (-0.04- -0.01)	<0.001
BMI (per 1 kg/m^2)	0.05 (0.01-0.08)	0.01	-0.01 (-0.04-0.02)	0.44
SBP (per 1 mmHg)	0.03 (0.02-0.04)	<0.001	0.02 (0.01-0.03)	<0.001
Alcohol status (yes vs. no)	-0.27 (-0.52- -0.01)	0.04	-0.004 (-0.21-0.21)	0.97
Smoking status (yes vs. no)	1.29 (0.75-1.83)	<0.001	0.17 (-0.03-0.38)	0.10
HTN (yes vs. no)	1.16 (0.85-1.47)	<0.001	0.60 (0.34-0.84)	<0.001
DM (yes vs. no)	1.79 (1.29-2.29)	<0.001	1.49 (1.18-1.80)	<0.001
CVDs (yes vs. no)	0.36 (-0.62-1.33)	0.47	0.88 (0.25-1.51)	0.01
Hemoglobin (per 1 g/dL)	0.10 (-0.01-0.20)	0.06	-0.19 (-0.26- -0.11)	<0.001
Fasting plasma glucose (per 1g/dL)	0.02 (0.01-0.03)	<0.001	0.01 (0.00-0.01)	<0.001
Albumin (per 1 g/dL)	-0.65 (-1.11- -0.19)	0.01	-1.76 (-2.04- -1.48)	<0.001
Total cholesterol (per 1 mg/dL)	0.005 (0.002-0.008)	0.01	-0.006 (-0.009- -0.004)	<0.001
CRP (per 1 mg/dL)	0.11 (-0.10-0.33)	0.29	0.07 (-0.10-0.23)	0.43
Proteinuria (yes vs. no)	0.64 (0.17-1.12)	0.01	0.88 (0.58-1.18)	<0.001

[a] The rate of decline in eGFR over time was determined using the least squares linear regression of eGFR over time; this was calculated from serial serum creatinine measured during the study period for each participant. The slope was expressed as the regression coefficient (mL/min/1.73 m^2/year).

[b] Linear regression analysis was performed with the decline rate of eGFR and each clinical factor.

Abbreviations: eGFR, estimated glomerular filtration rate; BMI, body mass index; SBP, systolic blood pressure; DM, diabetes mellitus; CVD, cardiovascular disease; CRP, C-reactive protein

Supplemental Table 2. Univariable Cox analysis for the risk of the development of eGFR <60 mL/min/1.73 m^2 according to quartiles of sCr levels

	**Female**		**Male**	
	**HR (95% CI)**	***P***	**HR (95% CI)**	***P***
Age (per 1 year)	1.14 (1.12-1.15)	<0.001	1.12 (1.10-1.14)	<0.001
Muscle mass (per 1kg)	0.99 (0.97-1.01)	0.45	0.97 (0.95-0.99)	0.02
BMI (per 1 kg/m^2)	1.06 (1.03-1.09)	<0.001	1.05 (1.01-1.10)	0.01
SBP (per 1 mmHg)	1.02 (1.01-1.03)	<0.001	1.03 (1.02-1.04)	<0.001
Alcohol status (yes vs. no)	0.66 (0.53-0.82)	<0.001	0.71 (0.54-0.93)	0.01
Smoking status (yes vs. no)	1.44 (0.98-2.090	0.06	0.88 (0.66-1.16)	0.35
HTN (yes vs. no)	2.88 (2.38-3.49)	<0.001	2.49 (1.91-3.25)	<0.001
DM (yes vs. no)	3.01 (2.32-3.92)	<0.001	3.05 (2.25-4.13)	<0.001
CVDs (yes vs. no)	1.71 (0.94-3.10)	0.08	2.79 (1.53-5.11)	0.01
Hemoglobin (per 1 g/dL)	1.17 (1.08-1.27)	<0.001	0.83 (0.75-0.92)	<0.001
Fasting plasma glucose (per 1g/dL)	1.01 (1.00-1.02)	<0.001	1.01 (1.01-1.02)	<0.001
Albumin (per 1 g/dL)	0.53 (0.37-0.75)	<0.001	0.39 (0.27-0.58)	<0.001
Total cholesterol (per 1 mg/dL)	1.01 (1.00-1.02)	<0.001	1.00 (0.99-1.01)	0.13
CRP (per 1 mg/dL)	1.06 (0.99-1.17)	0.21	1.11 (0.96-1.27)	0.15
Proteinuria (yes vs. no)	1.31 (1.06-1.62)	0.01	1.68 (1.18-2.38)	0.01

Abbreviations: eGFR, estimated glomerular filtration rate; sCr, serum creatinine; HR, hazard ratio; CI, confidence interval; BMI, body mass index; SBP, systolic blood pressure; DM, diabetes mellitus; CVD, cardiovascular disease; CRP, C-reactive protein

Supplemental Table 3. Risk the development of eGFR <60 mL/min/1.73 m^2 according to quartiles of sCr levels in different age groups [a]

	Age groups (years)					
	40-50		51-60		>60	
	HR (95% CI)	*P*	HR (95% CI)	*P*	HR (95% CI)	*P*
**Quartiles of sCr**						
**Female**						
**Q1 (n=2083)**	Reference		Reference		Reference	
**Q2 (n=1325)**	2.90 (0.96-8.75)	0.06	2.50 (1.31-4.76)	0.01	2.66 (1.73-4.09)	<0.001
**Q3 (n=862)**	5.48 (1.87-16.04)	0.01	5.11 (2.71-9.65)	<0.001	3.24 (2.05-5.11)	<0.001
**Q4 (n=684)**	12.06 (4.34-33.53)	<0.001	4.44 (2.23-8.84)	<0.001	4.09 (2.53-6.61)	<0.001
**Male**						
**Q1 (n=1457)**	Reference		Reference		Reference	
**Q2 (n=995)**	2.53 (0.46-13.92)	0.29	3.16 (1.04-9.63)	0.04	3.62 (1.82-7.22)	<0.001
**Q3 (n=956)**	4.94 (1.04-23.56)	0.04	9.05 (3.30-24.80)	<0.001	7.12 (3.68-13.79)	<0.001
**Q4 (n=1083)**	17.88 (4.11-77.81)	<0.001	12.46 (4.64-33.42)	<0.001	12.46 (6.38-24.34)	<0.001

[a] Models were adjusted for age, muscle mass, BMI, SBP, smoking and alcohol status, history of hypertension, DM, and CVDs, hemoglobin, fasting plasma glucose, serum albumin, total cholesterol, CRP, and proteinuria

Abbreviation: eGFR, estimated glomerular filtration rate; sCr, serum creatinine; HR, hazard ratio; CI, confidence interval; BMI, body mass index; SBP, systolic blood pressure; DM, diabetes mellitus; CVD, cardiovascular disease; CRP, C-reactive protein

Supplemental Table 4. Relative risk of CVD according to quartiles of sCr levels in female and male subject

	Model 1		Model 2		Model 3		Model 4	
	**OR (95% CI)**	***P***	**OR (95% CI)**	***P***	**OR (95% CI)**	***P***	**OR (95% CI)**	***P***
**Quartiles of sCr**								
**Female**								
**Q1 (n=2083)**	Reference		Reference		Reference		Reference	
**Q2 (n=1325)**	1.33 (0.74-2.38)	0.34	1.26 (0.62-2.54)	0.52	1.20 (0.59-2.44)	0.61	1.09 (0.52-2.27)	0.82
**Q3 (n=862)**	1.16 (0.58-2.32)	0.67	0.93 (0.40-2.17)	0.87	0.89 (0.38-2.09)	0.89	0.83 (0.35-2.00)	0.68
**Q4 (n=684)**	1.22 (0.58-2.56)	0.60	0.73 (0.27-2.02)	0.55	0.70 (0.25-1.92)	0.69	0.68 (0.24-1.94)	0.48
**Male**								
**Q1 (n=1457)**	Reference		Reference		Reference		Reference	
**Q2 (n=995)**	0.93 (0.4-1.83)	0.83	1.06 (0.43-2.63)	0.90	1.08 (0.43-2.69)	0.87	1.06 (0.42-2.65)	0.91
**Q3 (n=956)**	1.18 (0.62-2.24)	0.61	1.41 (0.61-3.25)	0.42	1.41 (0.61-3.27)	0.42	1.18 (0.49-2.84)	0.72
**Q4 (n=1083)**	1.67 (0.94-2.94)	0.07	1.92 (0.88-4.18)	0.10	1.87 (0.85-4.13)	0.12	1.68 (0.73-3.85)	0.22

Model 1: Unadjusted model
Model 2: Adjusted for age and muscle mass
Model 3: Adjusted for Model 2 + BMI, SBP, smoking and alcohol status, history of hypertension, DM, and CVDs
Model 4: Adjusted for Model 3 + hemoglobin, fasting plasma glucose, serum albumin, total cholesterol, CRP, and proteinuria
Abbreviation: CKD, chronic kidney disease; sCr, serum creatinine; OR, Odds ratio; CI, confidence interval; BMI, body mass index; SBP, systolic blood pressure; DM, diabetes mellitus; CVD, cardiovascular disease; CRP, C-reactive protein
